# Transient conformations in the unliganded FK506 binding domain of FKBP51 correspond to two distinct inhibitor-bound states

**DOI:** 10.1016/j.jbc.2023.105159

**Published:** 2023-08-12

**Authors:** Janet S. Anderson, David M. LeMaster, Griselda Hernández

**Affiliations:** 1 Department of Chemistry, Union College, Schenectady, New York, USA; 2 New York State Department of Health, Wadsworth Center, Albany, New York, USA

**Keywords:** FK506-binding proteins, conformational selection, induced fit, hydrogen exchange, NMR relaxation dispersion

## Abstract

Members of the FK506-binding protein (FKBP) family regulate a range of important physiological processes. Unfortunately, current therapeutics such as FK506 and rapamycin exhibit only modest selectivity among these functionally distinct proteins. Recent progress in developing selective inhibitors has been reported for FKBP51 and FKBP52, which act as mutual antagonists in the regulation of steroid hormone signaling. Two structurally similar inhibitors yield distinct protein conformations at the binding site. Localized conformational transition in the binding site of the unliganded FK1 domain of FKBP51 is suppressed by a K58T mutation that also suppresses the binding of these inhibitors. Here, it is shown that the changes in amide hydrogen exchange kinetics arising from this K58T substitution are largely localized to this structural region. Accurate determination of the hydroxide-catalyzed exchange rate constants in both the wildtype and K58T variant proteins impose strong constraints upon the pattern of amide exchange reactivities within either a single or a pair of transient conformations that could give rise to the differences between these two sets of measured rate constants. Poisson–Boltzmann continuum dielectric calculations provide moderately accurate predictions of the structure-dependent hydrogen exchange reactivity for solvent-exposed protein backbone amides. Applying such calculations to the local protein conformations observed in the two inhibitor-bound FKBP51 domains demonstrated that the experimentally determined exchange rate constants for the wildtype domain are robustly predicted by a population-weighted sum of the experimental hydrogen exchange reactivity of the K58T variant and the predicted exchange reactivities in model conformations derived from the two inhibitor-bound protein structures.

While it is clear that many physiologically significant proteins undergo transitions to transient alternative conformations, these transient states are often difficult to adequately characterize, both in terms of conformation and population. Such characterizations are yet more challenging for cases in which two alternative transient conformations can arise within a particular region of the protein. This circumstance is particularly relevant in structure-based drug design for flexible target proteins. When analyzed according to an induced-fit mechanism of binding, an initial weak binding event is then further analyzed in terms of the resultant ligand–protein interactions inducing a transition to a more stable selective complex. Alternatively, the same system can be analyzed in terms of two transient protein conformations, one well populated but weakly binding state and a second less populated but more tightly binding state. On the premise that such a conformational selection paradigm can provide a useful experimental guide, numerous laboratories have reported NMR relaxation studies on various small protein targets to identify sites within these structures that undergo conformational transitions in the ∼millisecond time frame, which give rise to observable exchange dynamic effects. One medically relevant system for such studies is the set of structurally homologous FK506-binding proteins (FKBPs), which have earned their name by virtue of the fact that the immunosuppressant drug FK506 rather indiscriminately inhibits many of the protein family members. Given that different members of this protein family regulate quite distinct physiological processes, such as cardiac/skeletal muscle contraction (FKBP12.6/FKBP12) (1, 2) and steroid hormone signaling (FKBP51/FKBP52) (3), there is a clear clinical need to develop drugs that selectively block individual members of this FKBP protein family. In particular, clinical mutations in FKBP51 have been associated with the onset of various stress-related diseases and mental disorders (4, 5), leading to its identification as a promising target for drug development (6, 7).

In an earlier report on the resonance assignments and ^15^N relaxation characteristics of the FK1 domain of the FKBP51 protein (8), we found evidence for a conformational transition occurring in the β_2_–β_3a_ hairpin that appears to be centered around the bifurcated hydrogen-bonding interactions involving Phe 67, which disrupts the otherwise regular antiparallel β-strand conformation of that hairpin. Following that work, Hausch *et al.* (9) reported a pair of lead compounds that bind selectively to FKBP51 but not to the closely homologous FKBP52 to yield the first such example of a designed selectivity among complementary members of the FKBP protein family. Their crystallographic analysis of the wildtype protein, both unliganded and in the iFit1- and iFit4-inhibited complexes (Fig. 1), indicated that the backbone conformation and hydrogen-bonding pattern significantly differ among all three structures in the β_2_–β_3a_ hairpin. In the iFit1- and iFit4-inhibited conformations, the phenyl ring of Phe 67 projects out into the solvent phase, whereas in the unliganded wildtype structure, that side chain remains internally buried beneath the tip of the long β_4_–β_5_ loop (10). In the iFit1- and iFit4-inhibited complexes, the difference in conformation for the β_2_–β_3a_ hairpin is primarily localized to the segment from Ser 62 to Phe 67 (Fig. 1*B*). Of particular relevance to the issue of drug selectivity with respect to FKBP52, Lys 58 of FKBP51 is substituted by threonine in FKBP52. When the K58T mutation is introduced into FKBP51, both the binding to the selective inhibitors (9) and the conformational transition(s) in the β_2_–β_3a_ hairpin of the unliganded protein (11) are suppressed. This effect presumably reflects the formation of a hydrogen bond between the side chains of Thr 58 and Ser 69 as is seen in the FKBP52 crystal structure, which would restrict the relative motion of the β_2_ and β_3a_ strands (12).

**Figure 1:**
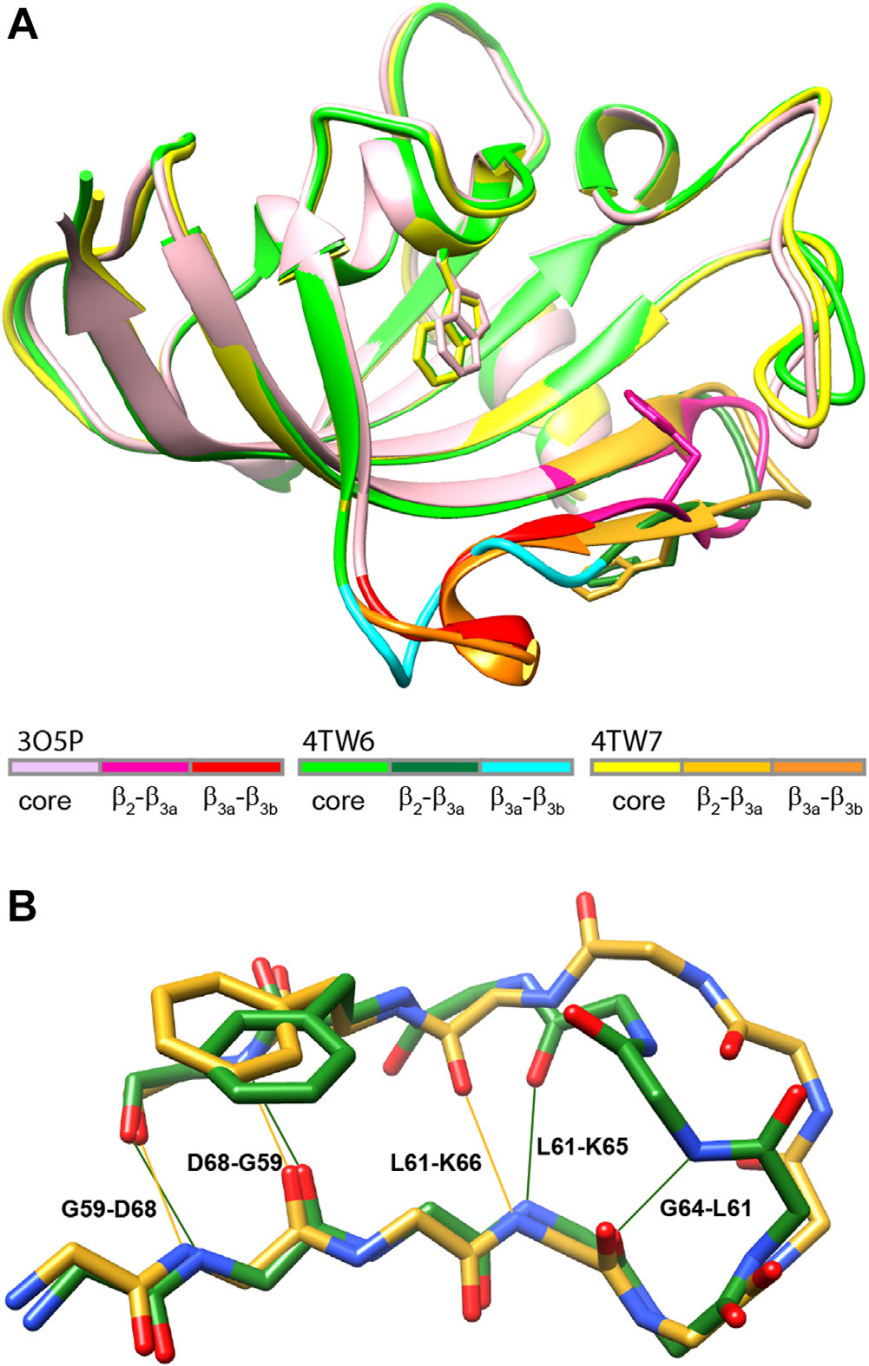
**The conformation of the β**_**2**_**–β**_**3a**_**hairpin of the FK1 domain of FKBP51 in apo and inhibited forms.***A*, illustration of the crystal structures of the unliganded wildtype (PDB code: 3O5P), iFit1-bound form (PDB code: 4TW6), and iFit4-bound form (PDB code: 4TW7). For each crystal structure, the protein core, β_2_–β_3a_ hairpin, and β_3a_–β_3b_ loop are color coded. Excepting Phe 67 in the β_2_–β_3a_ hairpin and Trp 90 at the base of the active site, all side-chain atoms have been removed for visual clarity. An expanded view of the β_2_–β_3a_ hairpin backbone is illustrated in *B* with the iFit1-bound form and the iFit4-inhibited structure similarly color coded. Interstrand hydrogen bonds are indicated. In these inhibitor-bound conformations, the side chain of Phe 67 is projected out into the solvent phase rather than being inserted into the protein interior beneath the long β_4_–β_5_ loop as seen in the unliganded wildtype structure. FKBP, FK506-binding protein; PDB, Protein Data Bank.

To further examine whether the ^15^N relaxation behavior for the unliganded FKBP51 domain is qualitatively consistent with a transition to a conformation resembling either the iFit1-inhibited (Protein Data Bank [PDB] code: 4TW6 (9)) or iFit4-inhibited (PDB code: 4TW7 (9)) crystal structures, the SPARTA+ program (13) was applied to predict the structure-based differential ^15^N chemical shifts for those two conformational states. Based upon that analysis, we proposed that this transient conformation of the β_2_–β_3a_ hairpin more closely resembles the iFit1-like conformation (11). In subsequent NMR relaxation studies, Hausch *et al.* (14) provided additional qualitative support for such a conformational selection mode of binding. Their ongoing drug design studies have indicated that the tighter binding iFit4-based analogs appear to be more fruitful for the development of selective derivatives (15). Crucial to the more detailed analysis of these interactions in terms of either an induced fit or a conformational selection model is the question of whether the unliganded protein adopts distinct transient conformations that closely resemble both the iFit1-bound and iFit4-bound states. This work presents the structure-based analysis of amide hydrogen exchange reactivity that provides such evidence.

Amide hydrogen exchange measurements have provided valuable insight into the conformational flexibility of proteins since the pioneering studies of Linderström–Lang nearly 70 years ago (16). As deduced by Manfred Eigen (17) and later experimentally verified by Molday and Kallen (18), simple peptides exhibit diffusion-limited Eigen kinetics in hydroxide-catalyzed exchange reactions. The reaction rate of an amide with hydroxide ion is attenuated from the diffusion limit (10^10.3^ M^−1^ s^−1^ at 25 °C (18, 19)) by a factor of *K*_i_/(*K*_i_ + 1), where *K*_i_ is the equilibrium constant for the transfer of a hydrogen from the amide to the hydroxide ion. Reflecting the fact that a neutral water molecule will rapidly quench the typically more strongly basic peptide anion, the lifetime of such an intermediate is only ∼10 ps (20, 21, 22), too short of a duration for significant conformational reorganization (23). As a result, the effective dielectric shielding for the transient peptide anion is constrained to a value near the optical limit, which has been estimated to be at least 2.5 within the protein interior (24). The brief lifetime of the peptide anion directly implies that the measured kinetic acidity of a peptide unit will be determined by the population average of the Boltzmann-distributed conformer reactivities and hence strongly dominated by the most acidic conformers (25). Given that the p*K*_a_ of water is 15.7, the relationship between the kinetic acidity and the thermodynamic acidity can be referenced to a log k_OH−_ value of 0 corresponding to a p*K*_a_ value of 26.

While these considerations imply that the exchange kinetics of a peptide hydrogen can be theoretically estimated from the electrostatic potential of the deprotonated peptide state (26, 27, 28), in the early 1970s when this opportunity was first recognized, robust atomic-level protein structure–based electrostatic modeling algorithms had not yet been developed. To circumvent this practical limitation, Englander (29) proposed that protein hydrogen exchange kinetics be analyzed in terms of “closed” and “open” states in which no hydrogen exchange occurs in the closed state and the exchange kinetics of the “open” state are defined as those of the corresponding random coil peptide. Unfortunately, the ease of reporting the apparent free energy of protein conformational equilibria in terms of the log ratio of hydrogen exchange rates has arguably discouraged adequate consideration of the underlying assumptions of that peptide normalization protocol (30). By thus discounting the structural factors that influence the acid–base reactivity of the backbone amide hydrogen groups, the often dramatic effects on the exchange kinetics that arise from differences in the local electrostatic environment can be left unexplained. The magnitude of the potential resultant errors is well illustrated by the fact that amide hydrogens within a single protein that are known to be well exposed to the solvent phase have been shown to exhibit a billion-fold range in hydrogen exchange kinetics (31). Unfortunately, the accurate structure-based prediction of protein ionizations remains challenging. Despite the simplistic physical assumptions of Poisson–Boltzmann continuum dielectric analysis, this approach remains a widely used method in predicting protein side-chain p*K*_a_ values. Analogous electrostatic calculations have similarly demonstrated that differential hydrogen exchange reactivities can be moderately well predicted for simple conformationally disordered peptides (26, 27, 28), for backbone amides that are conformationally exposed in crystal structures (22, 32), and for modeled protein conformational ensembles (25, 33).

A valuable illustration is provided from the analysis of simple dipeptides. The experimental hydroxide ion-catalyzed exchange rate constants for the nonpolar side-chain *N*-acetyl-[X-Ala]-*N*-methylamides and *N*-acetyl-[Ala-Y]-*N*-methylamides lie within a 10-fold range (30). The Protein Coil Library (34) was used to generate a conformational ensemble of these dipeptides that was anticipated to realistically model a Boltzmann-weighted distribution. For each such dipeptide, Poisson–Boltzmann continuum dielectric calculations predicted hydrogen exchange reactivities for the individual conformers that spanned a million-fold range (28). Yet the ensemble averaging of these conformer reactivities accurately predicted the experimental hydrogen exchange rate constants for each of the nonpolar dipeptides with a correlation coefficient of 0.91. A qualitatively similar conclusion has been drawn from the analysis of experimentally restrained protein conformational ensembles that had previously been designed to mimic a Boltzmann distributed sampling. In comparison to electrostatic calculations on the solvent-exposed backbone amides in the ubiquitin crystal structure, a corresponding analysis of the 144 conformations in the NMR relaxation–restrained molecular dynamics ensemble of ubiquitin 2NR4 (35) yielded a nearly twofold reduction in the standard deviation with respect to the experimental hydrogen exchange data (25).

The FKBP51 domain offers an exceptionally promising test system for examining the degree to which structure-based peptide hydrogen acidity analysis can efficiently model the fractional concentration for multiple transient minor conformational states in a fashion that is arguably inaccessible by any other currently available experimental approach. This study examines how continuum dielectric predictions of the peptide acidities derived from the iFit1-liganded 4TW6 and iFit4-liganded 4TW7 crystal structures (Fig. 1) can be used to robustly model transient conformational states that give rise to the substantial differences in the experimental hydrogen exchange kinetics within the β_2_–β_3a_ hairpin for the wildtype and K58T variant domains of FKBP51.

## Results

### Differential hydrogen exchange reactivity in the FKBP51 domain

For more rapidly exchanging protein amides, the rate at which selectively excited water-bound protons exchange onto backbone amide sites can be directly monitored by NMR methods. Utilizing protein samples in which all nonexchangeable hydrogen positions are perdeuterated for enhanced spectral resolution and sensitivity, amide exchange rates from less than 0.5 s^−1^ to 70 s^−1^ can be accurately determined (36). Typically, peptide hydrogen exchange is exclusively hydroxide ion catalyzed for pH values above 4. As previously reported for various well-studied proteins, such as ubiquitin, rubredoxin, chymotrypsin inhibitor 2, and FKBP12, the vast majority of backbone amides exhibit log exchange rates that follow a simple linear pH dependence (22). By carrying out a series of measurements spanning the range of pH 6 to 10, the hydroxide ion–catalyzed exchange rate constants can be readily determined over the range of 10^4^ to 10^9^ M^−1^ s^−1^. Since many of the residues that exchange within the pH range between 6 and 10 provide accurate measurements in two or more of the pH values sampled, utilizing such crosscalibration can provide well-integrated results across the full pH range examined. Typically, it is only a subset of histidine side chains that undergo significant charge state titration within this pH range. In such instances, the determination of that side-chain *p*K value from the associated NMR chemical shift effects can potentially be used to derive the pH-independent log k_OH−_ rate constants for the nearby backbone amides that are perturbed by the ionization (22).

**Figure 2:**
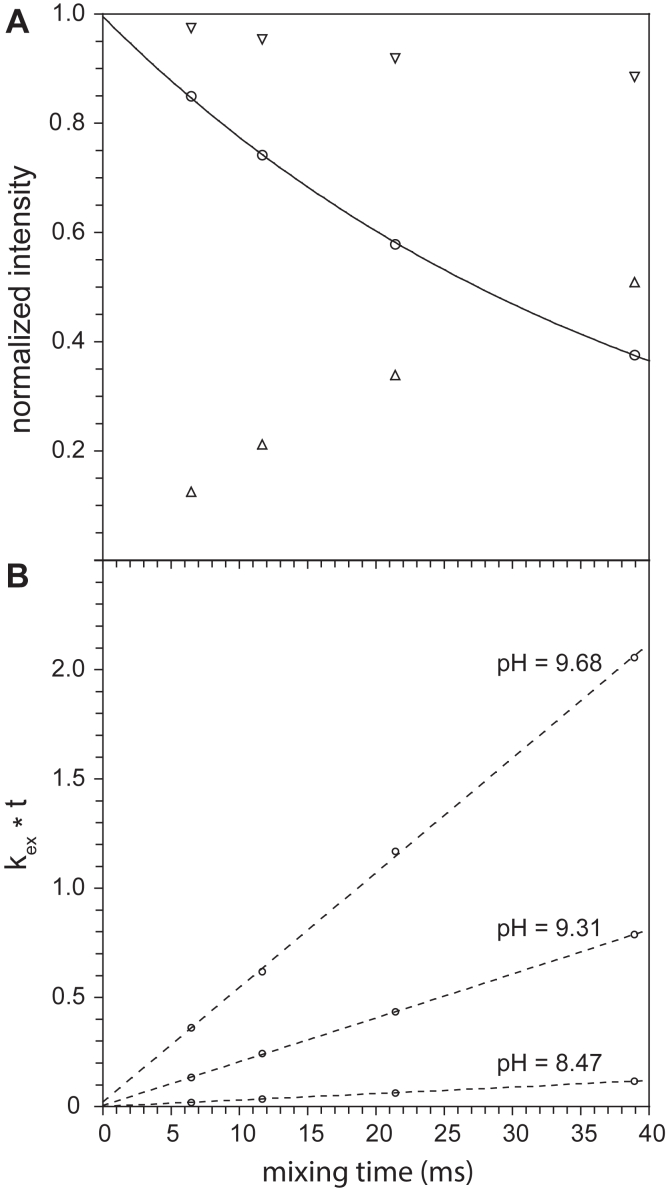
**CLEANEX-PM mixing time dependence of amide hydrogen exchange for Val 40 of the wildtype FKBP51 domain.***A*, shown is the normalized cross-peak intensities for the standard CLEANEX-PM-FHSQC experiment (△) and the hard pulse reference experiment in which the e-PHOGSY component is replaced with a high power 90° ^1^H pulse (▽) for Val 40 at pH 9.31. The time constant for the derived exponential decay (Ο) is equal to ρ + *k*_ex_. For each of the three pH samples analyzed, consistent estimates for *k*_ex_ were derived across the range of CLEANEX-PM mix times (*B*). FKBP, FK506-binding protein.

As the ability to quantify the populations for the transient protein conformations of FKBP51 is strongly dependent upon the quality of the hydrogen exchange data, an optimized analysis protocol was applied. The widely used CLEANEX-PM experiment (37, 38) applies an e-PHOGSY component (39) to selectively excite the ^1^H_2_O resonance. During the subsequent mixing period, ^1^H spins from the excited ^1^H_2_O resonance exchange onto backbone amide sites during which time the CLEANEX-PM pulse sequence selects for the signal from these chemically exchanged protons. As illustrated for Val 40 of the wildtype FKBP51 domain (Fig. 2*A*), the resultant buildup curve deviates from linearity at relatively short mix times. By substituting a high-power 90° ^1^H pulse for the e-PHOGSY component, a reference spectrum can be obtained, which monitors the decay of the spin magnetization for the protons that are initially bound to the amide positions. The difference between these two signals exponentially decays with a time constant corresponding to the sum of the spin relaxation rate for the ^1^H^N^ resonance during the CLEANEX-PM mix time (ρ) and the chemical exchange rate (*k*_ex_) (Fig. 2*A*). The transverse magnetization of the CLEANEX-PM signal is then given by (40):

(1) $$M_{y}\left(t\right)=\left[\frac{k_{ex}}{\left(\rho +k_{ex}\right)}\right]*\left\{1-\exp\left[-\left(\rho +k_{ex}\right)t\right]\right\}$$

The analysis of the CLEANEX-PM and hard pulse reference spectra enables an independent determination of *k*_ex_ at each mix period from which the standard closed form linear least squares estimation of the statistical uncertainty in the *k*_ex_ values can be obtained (Fig. 2*B*). Under the condition of equivalent peak intensity noise across the mix time series, the individual deviations from the best fit line and the distribution of sampling times analytically determine the statistical uncertainty in the resultant *k*_ex_ values. As illustrated by the CLEANEX-PM data of Val 40 at pH 8.47 (Fig. S1), the sensitivity of these experiments is sufficient to yield robust rate estimates across a 100-fold range for these protein samples.

**Figure 3:**
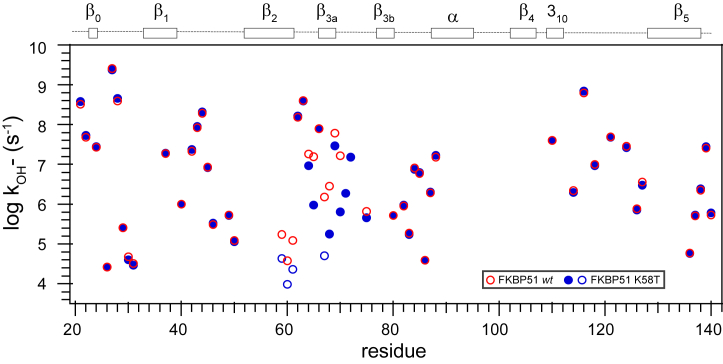
**Hydrogen exchange rate constants for the wildtype and K58T variant of the FKBP51 domain.** Residues for which log k_OH−_ values were determined by CLEANEX-PM measurements in the wildtype domain (*red circle*) are compared with analogous data from the K58T variant (*blue*). An rmsd of 0.04 was observed between these two datasets for the monitored residues that lie outside the segment between Gly 59 and Glu 75. The diameter of the symbols closely approximates the 95% confidence limits for these data. For four slowly exchanging residues within the β_2_–β_3a_ hairpin of the K58T variant (Gly 59, Lys 60, Leu 61, and Phe 67 [*blue circles*]), hydrogen–deuterium isotope exchange-in experiments were used to determine the log k_OH−_ values. The numbering of the β-strand segments reflects that of the canonical FKBP12 structure. FKBP, FK506-binding protein.

The utility of this level of precision was illustrated in the comparison between the CLEANEX-PM data for the wildtype FKBP51 domain and K58T variant (Fig. 3 and Table S1). The majority of the residues that exchange too slowly to be detected by these CLEANEX-PM experiments lay within the regular secondary elements of the protein. For the monitored residues that lay outside the β_2_–β_3a_ hairpin (Gly 59–Asp 68) and the adjacent β_3a_–β_3b_ loop (Ser 69–Glu 75), the rmsd between the log k_OH−_ rate constants from the wildtype and the K58T variant protein is 0.04. Since 10^0.04^ corresponds to a factor of 10% and more than five orders of magnitude are being monitored in these peptide acidity measurements, variations in the exchange reactivity of typical backbone peptide hydrogens can be reliably distinguished over a million-fold range. This experimental data directly indicates that any differences in the conformational flexibility exhibited by the wildtype and K58T variant proteins in regions outside the β_2_–β_3a_ hairpin and β_3a_–β_3b_ loop are constrained to be effectively silent to hydrogen exchange dynamics.

In notable contrast, most of the residues between Gly 59 and Asp 68 in the β_2_–β_3a_ hairpin exhibit considerably larger differential log k_OH−_ values. This is also the case for the log k_OH−_ values in the β_3a_–β_3b_ loop. Unfortunately, the CLEANEX-PM exchange analysis is rendered comparatively unreliable within this loop because of not only the significant line-broadening effects of the microsecond–millisecond conformational dynamics in this segment (8) but also the effects of the His 71 side chain. Its ionization with a *p*K of ∼6.3 introduces a varying level of the distinct electrostatic environments arising from either the positive imidazolium or neutral imidazole side-chain states across the pH range in which the hydrogen exchange kinetics of the nearby residues are observed, thus severely complicating the extraction of pH-independent log k_OH−_ values.

The log k_OH−_ rate constants and their estimated uncertainties for the individual residue data were used to estimate the aggregate uncertainty in the *k*_ex_ analysis discussed previously. For the wildtype and K58T variant domains, overall rmsd values of 0.029 and 0.033 were obtained for the log k_OH−_ values across the full set of individual *k*_ex_ analyses (Table S1). For those residues that gave rise to robust *k*_ex_ determinations at multiple pH values, the resultant differences in the corresponding log k_OH−_ values were used to derive an estimate of the uncertainty that arose during the establishment of the pH calibration across the sample set. The resultant intersample pH-dependent uncertainty estimates were 0.027 and 0.032 for the wildtype and K58T variant domains, respectively. Although undoubtedly the experimental uncertainty in this pH-dependent intersample comparison is not statistically independent of the uncertainty estimates from individual CLEANEX-PM experiments, treating these two uncertainty estimates as statistically independent provides a reasonable upper bound to the overall uncertainty in this hydrogen exchange analysis. Under that assumption, the average uncertainties in the log k_OH−_ values for the wildtype and K58T variant domains are 0.040 and 0.046, respectively. As supported by the similar level of variance seen in the direct comparison of the experimental data between the two proteins (Fig. 3), these levels of uncertainty in the experimental log k_OH−_ values were applied to the Monte Carlo analyses discussed later.

### Differential conformational dynamics in the β_2_–β_3a_ hairpin and β_3a_–β_3b_ loop

Given that differential hydrogen exchange behavior is observed in both the β_2_–β_3a_ hairpin and the adjacent β_3a_–β_3b_ loop, the use of these peptide acidity data to analyze conformational transitions within the β_2_–β_3a_ hairpin presupposes that the energetics of those transitions are effectively independent of the conformational dynamics occurring within the β_3a_–β_3b_ loop. Previously published magnetic field strength–dependent ^15^N relaxation measurements on the FKBP51 domain have demonstrated that introduction of the K58T mutation appeared to quench the microsecond–millisecond conformational dynamics around Phe 67 in the β_2_–β_3a_ hairpin, but only partially attenuated the dynamics within the β_3a_–β_3b_ loop (11). These earlier results suggested that the conformational dynamics of the β_2_–β_3a_ hairpin may be at least partially uncoupled from that of the β_3a_–β_3b_ loop. To further clarify this issue, we carried out a series of ^1^H relaxation dispersion measurements, which in favorable circumstances can characterize the effective rate of the monitored conformational exchange. While ^15^N CPMG (Carr–Purcell–Meiboom–Gill) relaxation dispersion experiments have been previously reported for the wildtype FKBP51 domain (14), the much lower Larmor frequency of the ^15^N nucleus limited those experiments to a maximum sampling frequency of 1 kHz, which proved to be too slow to adequately characterize the conformational dynamics of these regions. By applying ^1^H^N^ constant time (CT)-CPMG relaxation dispersion measurements (41), the maximum sampling frequency was extended to 8 kHz. When feasible, the use of ^2^H,^15^N enriched protein samples is particularly beneficial for such measurements as deuterium substitution at nonamide sites serves to eliminate extraneous ^1^H–^1^H interactions that can seriously compromise data analysis for this class of experiments. While modifications of the standard relaxation dispersion pulse sequence can help suppress such undesired effects in nondeuterated samples (42), deuterium substitution provides a particularly robust approach.

Dynamical parameters were obtained for all residues that exhibited CPMG field-dependent variation in R_2(eff)_ greater than 2.0 s^−1^ for the wildtype FKBP51 domain and the K58T variant, based upon the collection of two independent datasets for each protein (Tables S2 and S3). For both proteins, the data from all residues in the β_2_–β_3a_ hairpin and the β_3a_–β_3b_ loop were well fitted by the two-parameter Luz–Meiboom fast limit two-site exchange model (43). A significant limitation of this dynamical model is that, while the refocusing frequency dependence of the CPMG signal provides the conformational exchange rate *k*_ex_, the populations of the two exchanging sites cannot be directly deconvoluted from the (unknown) chemical shift difference between those sites, resulting in an unresolved factor p_a_p_b_∗(ω_Ha_−ω_Hb_)^2^. The more detailed three-parameter Carver–Richards dynamical model can provide for estimation of the site populations from relaxation dispersion data that significantly deviate from the constrained set of curves that are consistent with the Luz–Meiboom model (44). However, utilizing the Akaike’s information criterion for model selection as implemented in the RELAX 5.0.0 NMR data analysis package (45), the Carver–Richards model was statistically rejected for all relaxation dispersion curves within this region of the protein, although several residues within the long β_4_–β_5_ loop did yield selection of this more complex model.

**Figure 4:**
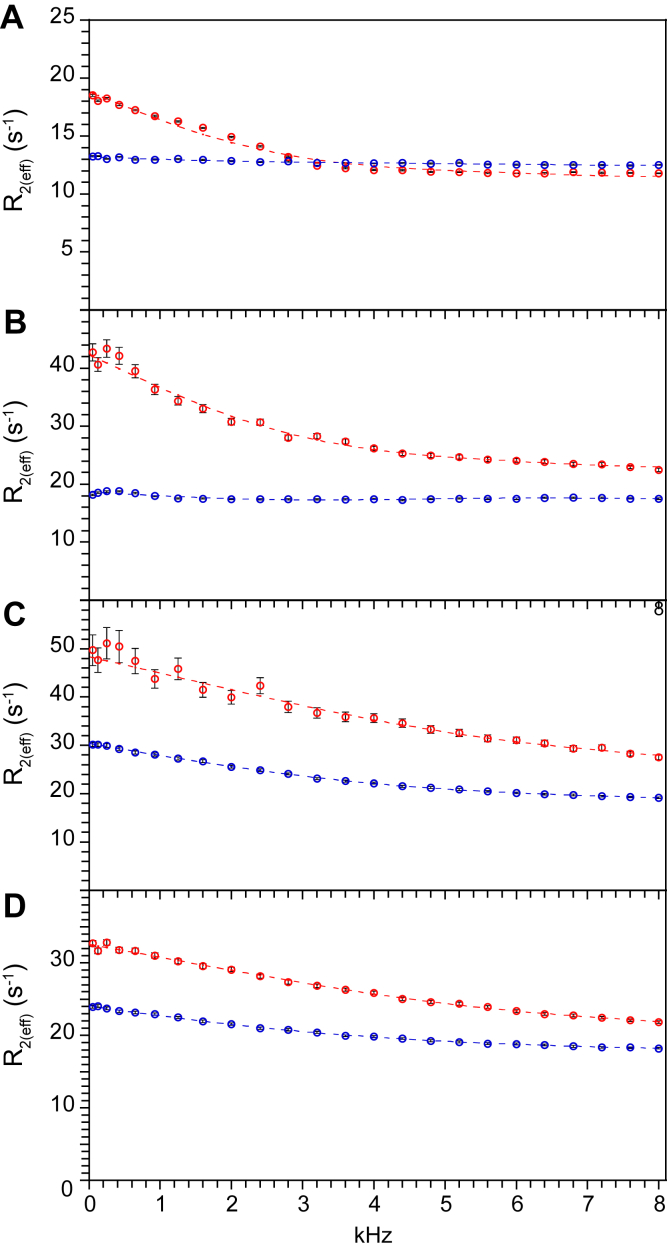
^**1**^**H**^**N**^**CT-CPMG relaxation dispersion data for selected residues in the β**_**2**_**–β**_**3a**_**hairpin and the β**_**3a**_**–β**_**3b**_**loop.** Over the refocusing frequency of 50 Hz to 8 kHz, for Lys 66 (*A*) and Asp 68 (*B*) in the β_2_–β_3a_ hairpin, the K58T substitution (*blue*) strongly suppresses the dynamical line broadening seen in the wildtype protein (*red*). In contrast, substitution of the K58T variant only partially suppresses the dynamics of Asp 72 (*C*) and Asn 74 (*D*) within the β_3a_–β_3b_ loop, resulting in a roughly twofold lower amplitude of dynamical line-broadening effect in the K58T variant. All data were fitted to the Luz–Meiboom fast limit two-site exchange model (43). CT-CPMG, constant time Carr–Purcel–Meiboom–Gill pulse sequence.

Within the β_2_–β_3a_ hairpin of the wildtype FKBP51 domain, Lys 66 and Asp 68 exhibited the two most intense ^1^H^N^ relaxation dispersion responses, similar to their predominance in the ^15^N relaxation analysis of conformational exchange line broadening for this segment (11). For both these residues, the relaxation dispersion response is strongly suppressed in the K58T variant (Fig. 4, *A* and *B*), in clear contrast to Asp 72 and Asn 74 in the β_3a_–β_3b_ loop for which the K58T substitution induces only a partial suppression of dynamics (Fig. 4, *C* and *D*). Qualitatively similar relaxation dispersion kinetics were observed for His 71 and Arg 73, although in both instances, a substantial proportion of the R_2(eff)_ values were above 50 s^−1^ where the enhanced magnitude of conformational line broadening substantially degraded the signal intensities, compromising the corresponding dynamical analysis. It may be noted that, even by extending the spectral dynamic range up to 8 kHz, the dynamics of these β_3a_–β_3b_ loop residues are near the practical upper limit for characterization by these ^1^H^N^ relaxation dispersion measurements.

**Figure 5:**
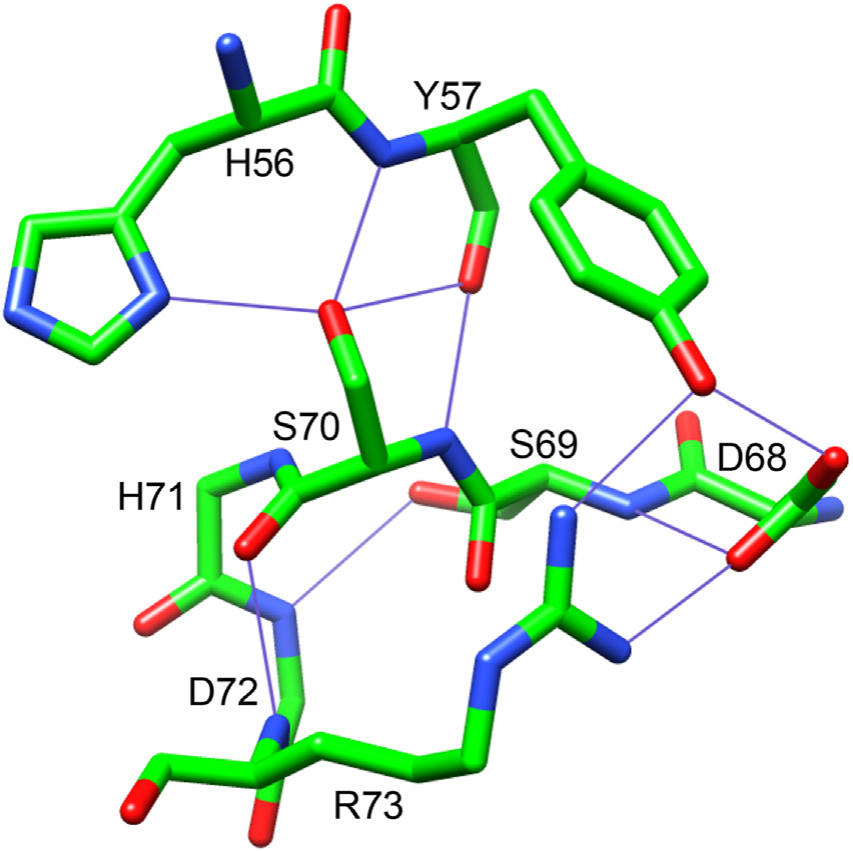
**Structural interactions of the β**_**3a**_**–β**_**3b**_**loop with Tyr 57 and Phe 68 in the wildtype FKBP51 domain.** Tyr 57 forms multiple hydrogen-bonding interactions with the β_3a_–β_3b_ loop through its backbone carbonyl and amide as well as its side-chain phenolic group (Protein Data Bank code: 3O5P). That phenolic group also hydrogen bonds to the carboxylate group of Asp 68 as does the guanidinium side chain of Arg 73. The side chains of His 71 and Asp 72 were deleted for visual clarity. FKBP, FK506-binding protein.

The β_3a_–β_3b_ loop residues Asp 72 and Asn 74 yielded quite similar dynamics in the wildtype domain with *k*_ex_ values of 3.09 (±0.35) × 10^4^ s^−1^ and 3.05 (±0.14) × 10^4^ s^−1^, respectively. The dynamics for these two residues are 2.4-fold faster than the kinetics observed for the β_2_–β_3a_ hairpin residue Lys 66, strongly indicating that distinct conformational transitions are driving the observed relaxation effects. Indeed, with one exception, all other residues outside the β_3a_–β_3b_ loop that exhibit relaxation dispersion kinetics have *k*_ex_ values that are more than twofold slower than Asp 72 and Asn 74. While Tyr 57 resides near the center of the β_2_ strand, its ^1^H^N^ resonance exhibits exchange dynamics that are quite similar to what was observed within the β_3a_–β_3b_ loop (Table S2). A plausible structural basis for this apparent dynamical coupling can be found in the three hydrogen-bond interactions that Tyr 57 forms with residues in the β_3a_–β_3b_ loop (Fig. 5). In this regard, it should be noted that the side-chain carboxylate of Asp 68 also forms salt bridge–hydrogen bond interactions with the guanidinium group of Arg 73 and the phenolic hydrogen of Tyr 57. All these interactions are preserved in crystal structures of the evolutionarily related FKBP12 (PDB code: 2PPN (46)) and FKBP52 FK1 domain (PDB code: 4LAV (12)).

Reflecting the presence of these direct structural interactions, the relaxation dispersion curves were examined for potential evidence of dynamical coupling between Asp 68 and the β_3a_–β_3b_ loop. In contrast to Lys 66, Asp 68 exhibits a more complex relaxation dispersion curve, which was closely reproduced upon repetition of the experiment. Such deviations from the predicted functional form suggest that the assumed fast limit two-site exchange model may be overly simplistic for this residue. At lower CPMG frequencies, the fitted Asp 68 relaxation dispersion curve appears to decay at a qualitatively similar rate as that of Lys 66. On the other hand, at CPMG frequency values above 4 kHz, the relaxation dispersion curve of Lys 66 is relatively flat, whereas that of Asp 68 continues to significantly decrease with a slope that is not dissimilar to what was seen for Asp 72 and Asn 74. These data suggest that Asp 68, but not Lys 66, is dynamically sensitive to a higher frequency transition. The potential implication of dynamical coupling between Asp 68 and the β_3a_–β_3b_ loop raises the possibility that the hydrogen exchange reactivity of Asp 68 might be similarly perturbed in a fashion not reflected by the rest of the residues within the β_2_–β_3a_ hairpin. In contrast to Asp 68, the other residues of the β_2_–β_3a_ hairpin would appear to be dynamically uncoupled from the internal mobility exhibited in the β_3a_–β_3b_ loop.

In comparing the relaxation dispersion dynamics between the wildtype and K58T domains, for both Asp 72 and Asn 74, the *k*_ex_ values decrease by 30% in the K58T protein (Tables S2 and S3). Quite similar decreases were observed for Tyr 57 and Glu 75 as well. A much larger effect was observed for the factor p_a_p_b_∗(ω_Ha_−ω_Hb_)^2^, which was shifted by 2.9 (±0.5) for these four residues. In contrast, for residues outside the β_2_–β_3a_ hairpin/β_3a_–β_3b_ loop region, the differential dynamical effects of the K58T substitution are appreciably smaller. Within that set, among the residues that are optimally fitted by the simpler fast limit two-site exchange model for both the wildtype and K58T variant domains, there is essentially no effect of the K58T substitution on the p_a_p_b_∗(ω_Ha_−ω_Hb_)^2^ factor (1.0 [±0.17]), consistent with a negligible difference in state population for the conformational dynamics that give rise to those relaxation dispersion effects. In a similar fashion, for the residues (all within the long β_4_–β_5_ loop) that consistently yielded the three-parameter Carver–Richards dynamical model in either the wildtype or K58T variant domains, the predicted populations for the minor state were statistically indistinguishable among all such residues. These results are consistent with the conclusion from the previous section that, beyond the β_2_–β_3a_ hairpin/β_3a_–β_3b_ loop region, the differential conformational samplings of the wildtype and K58T variant proteins do not give rise to significant differences in the populations of hydrogen exchange–active conformations (Fig. 3).

### Peptide acidity analysis of single and dual transient state models as applied to the β_2_–β_3a_ hairpin

The ability to reliably predict the hydrogen exchange reactivity of a model peptide conformation provides an experimental basis for characterizing the conformation of transient metastable states. Such analysis is comparatively straightforward for the circumstance in which only a single transient state contributes to the differences in exchange behavior between two experimentally characterized protein conformations. For systems in which numerous backbone amides report upon such differences in exchange behavior, it becomes potentially feasible to simultaneously characterize the contributions from two distinct transient states. Since their introduction 40 years ago (47), finite difference Poisson–Boltzmann continuum dielectric methods have remained a widely used approach to predicting protein side-chain p*K*_a_ values based upon atomic resolution structural data. Despite considerable effort to further improve the accuracy of these predictions, large-scale comparisons to experimental titration data indicate that the average reliability of such calculations remains at approximately 0.55 for surface-exposed side chains (48). While the continuum dielectric assumption is unquestionably simplistic, the practical reality is that accurate all-atom calculations of dielectric relaxation phenomena remains challenging even for simple solvent systems (49). In operational terms, the “internal dielectric” value that is invoked in the standard Poisson–Boltzmann prediction of side-chain ionizations must function, in part, to approximate the reorientations of both formal and partial charges within the protein that occur during the lifetime of a side-chain charge state, typically on the order of microseconds or longer. As noted previously, calculation of the electrostatic potential for the peptide anion is markedly facilitated by the very short lifetime of this state (∼10 ps), which severely curtails the range of conformational reorganization that can efficiently contribute to dielectric shielding. On the other hand, since the magnitude of the predicted solvation free energies is approximately inversely proportional to the assumed internal dielectric constant, the comparatively low internal dielectric value of 3 that has been found to be optimal for peptide anion calculations markedly exacerbates the effects of any errors in the structural modeling (22). On the basis of the far smaller set of experimental comparisons currently available for calibrating the accuracy of protein peptide acidity calculations, our previous work has derived estimates of 0.5 for the uncertainty in the log k_OH−_ values for backbone amide hydrogens that are solvent exposed in at least half of the sampled molecular dynamics trajectory frames, whereas that estimated uncertainty rises to around 0.7 for those amide hydrogens that are exposed in more than 1% but less than 50% of those frames (33).

**Figure 6:**
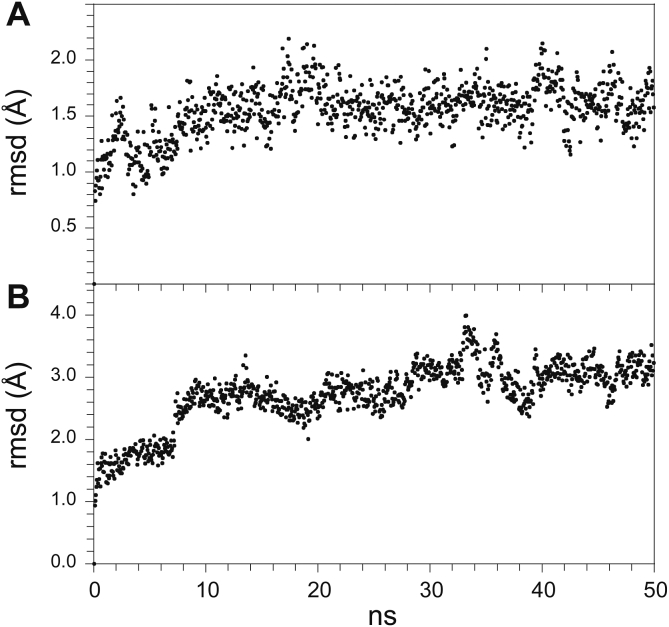
**Main-chain heavy atom rmsd values in β**_**2**_**–β**_**3a**_**hairpin for simulation trajectories on the 4TW6- and 4TW7-based structural models.** For the 4TW6- (*A*) and 4TW7-based (*B*) models, the rmsd values with respect to the initial frame were sampled at 50 ps intervals and averaged. In each simulation, the initial frames deviated from the reference X-ray structures by less than 0.5 Ǻ.

Distinct from the issue of accuracy in the continuum dielectric calculations of peptide acidity is the challenge of whether a given set of protein conformations adequately represents a Boltzmann distribution. While the Protein Coil Library has been successfully used to represent such a distribution for small model peptides (28), confident modeling of thermodynamic distributions derived from simulations based on protein crystal structures is less straightforward. The uncertainty estimates for log peptide acidity constants listed previously (*i.e.*, 0.5 and 0.7) were based upon distributions derived from molecular dynamics simulations and thus include errors arising from inadequate conformational sampling as well as inaccuracies in the electrostatic predictions of peptide conformer acidities. Following the protocol of these earlier studies (33), the conformational neighborhoods for the proposed transient states of the FKBP51 domain were derived from the iFit1-liganded 4TW6 and iFit4-liganded 4TW7 crystallographic determinations. For both crystal structures, a series of four 50 ns CHARMM36 molecular dynamics production runs were carried out following removal of the inhibitor and initial equilibration of the apoconformation (50). For each such trajectory, the heavy atom backbone rmsd values exhibited a rapid divergence from the first sampled frame and then reached an apparent plateau value during the second half of the simulation. As averaged over the four production runs, the plateau value for the 4TW7-derived simulations was nearly twice as large as that for the 4TW6-derived simulations (Fig. 6). Relevant to this differing behavior was the observation that the four intrahairpin hydrogen bonds between backbone atoms in the 4TW6-derived simulations (Fig. 1*B*) were preserved in each of those four 50 ns simulations. In contrast, for all four of the 4TW7-derived simulations, one of the three such intrahairpin main-chain hydrogen bonds (Leu 61 H^N^–Lys 66 O) was rapidly disrupted, giving rise to a markedly increased flexibility in the expanded loop region of the hairpin.

DelPhi (51) electrostatic calculations were carried out on trajectory frames sampled at 50 ps intervals. The population average of the peptide acidities derived from each of the sampled trajectory frames was used to predict log k_OH−_ values for each residue position. These log k_OH−_ values were then averaged across the four independent molecular simulations (Table 1). The experimental peptide hydrogen exchange rate constants for the entire β_2_–β_3a_ hairpin in the wildtype protein are either essentially equal to (Ser 62, Asn 63, and Lys 66) or greater than those of the K58T variant (Table 1). If protein conformations derived from the iFit-1 and/or iFit-4 liganded crystal structures are to explain those instances of enhanced hydrogen exchange kinetics in the wildtype FKBP51 domain, then the exchange reactivity in those weakly populated transient conformations must be substantially higher than what is experimentally observed in the wildtype protein. Indeed, with the exception of Asp 68, each residue in the β_2_–β_3a_ hairpin that exhibited a substantial differential in the experimental log k_OH−_ values for the wildtype and K58T proteins also yielded a DelPhi-predicted log k_OH−_ value for either the 4TW6- or 4TW7-derived conformation that markedly exceeds the experimental value for the wildtype FKBP51 domain. In contrast, not only does the amide hydrogen of Asp 68 fail to become solvent accessible during the molecular dynamics sampling of 4TW6-derived conformations, the predicted log k_OH−_ value for the 4TW7-derived conformation is nearly identical to the experimental value for the wildtype protein. As a result, an implausibly high population for the 4TW7-derived conformation would be required to match the experimental data. In light of the evidence for a dynamic coupling between Asp 68 and the residues of the β_3a_–β_3b_ loop discussed in the previous section, the subsequent analysis of hydrogen exchange reactivity in the β_2_–β_3a_ hairpin was limited to the segment of Gly 59 to Phe 67.

**Table 1:** Experimental and predicted log k_OH−_ values in the β_2_–β_3a_ hairpin

Residue	wt^a^	K58T^a^	4TW6^b^	Access^c^	4TW7^b^	Access^c^
Gly 59	5.25	4.64^d^	5.98	0.29	6.73	0.07
Lys 60	4.58	3.99^d^	3.67	0.03	7.86	0.12
Leu 61	5.09	4.36^d^		<0.01	7.64	0.44
Ser 62	8.19	8.21	7.39	0.18	8.71	0.31
Asn 63	8.61	8.60	8.99	0.60	9.51	0.79
Gly 64	7.26	6.97	7.01	0.02	9.02	0.49
Lys 65	7.19	5.98		<0.01	9.66	0.99
Lys 66	7.90	7.90	8.33	0.99	8.71	0.79
Phe 67	6.19	4.71^d^	6.76	0.99	7.51	0.96
Asp 68	6.37	5.27		<0.01	6.41	0.15

a Experimental log k_OH−_ values.

b Apoprotein trajectory-based DelPhi predictions.

c Fraction of trajectory frames with solvent exposure >0.5Ǻ^2^.

d Data obtained from isotope exchange-in measurements.

The three-state model for the amide hydrogen exchange for each residue of the β_2_–β_3a_ hairpin in the wildtype FKBP51 domain is given by:

(2) $$10^{\left[\log\;k_{OH}\left(wt\right)\right]}=\left(1-p_{A}-p_{B}\right)*10^{\left[\log\;k_{OH}\left(K58T\right)\right]}+p_{A}*10^{\left[\log\;k_{OH}\left(A\right)\right]}+p_{B}*10^{\left[\log\;k_{OH}\left(B\right)\right]}$$

**Figure 7:**
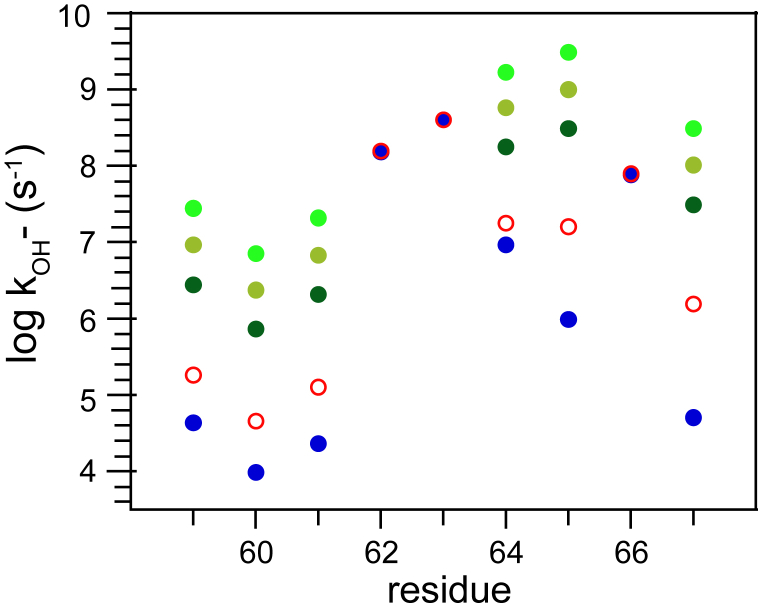
**Two-state population analysis of the experimental wildtype FKBP51 domain and K58T variant hydrogen exchange.** Assuming a single transient conformation at population values of 5% (*dark green*), 1.5% (*olive*), or 0.5% (*green*), the corresponding hydrogen reactivities for that transient conformation were determined by applying the experimental hydrogen exchange data to Equation 2 with p_B_ set to zero. For residues Ser 62, Asn 63, and Lys 66, the similarity in the exchange behavior for the wildtype protein (*red circle*) and K58T variant (*blue*) precludes a reliable assessment of the exchange reactivity within the transient conformation. FKBP, FK506-binding protein.

The fashion in which the experimental hydrogen exchange data can constrain the population values for the minor conformations is most readily illustrated for the circumstance in which only a single minor species contributes to the differential hydrogen exchange behavior such that p_B_ is set to zero in Equation 2. For any population value of the single minor state, the corresponding log k_OH−_ values for that minor state are explicitly determined from the experimental hydrogen exchange data. As applied to the experimental data for the wildtype and K58T variant domains (Table 1), assuming minor population values for p_A_ of 5%, 1.5%, or 0.5% yield correspondingly sets of deduced log k_OH−_ values for any experimentally consistent transient conformation of residues Gly 59 to Phe 67 (Fig. 7). With the experimental uncertainties of 0.040 and 0.046 in the differential log k_OH−_ values for the wildtype and K58T variant, respectively, a similar level of uncertainty will apply to the predicted log k_OH−_ values for all residues in which the transient conformation provides the dominant contribution to the observed hydrogen exchange kinetics. In this particular example, six residues give rise to significant differential hydrogen exchange behavior. As a result, the nominal six-dimensional search over the log k_OH−_ values for these residues in the single minor conformation analysis is reduced to a one-dimensional search over the population value of that minor state p_A_.

A model peptide conformation that yields hydrogen exchange reactivities consistent with the analysis illustrated in Figure 7 is a necessary but not sufficient condition for explaining the observed wildtype FKBP51 domain measurements on the basis of a single transient state. The hydrogen exchange reactivities of that transient state must reflect a Boltzmann-averaged ensemble that is structurally consistent with its covalent attachment to the protein domain. In practical terms, the stringency of these constraints upon the experimentally compatible model peptide conformations is appreciably weakened by the comparatively low precision with which the Boltzmann-averaged conformer reactivities can be predicted from structure-based calculations. That said, the log k_OH−_ prediction for each of these six residues provides an independent constraint upon the value of p_A_. As a result, the ability of these comparatively inaccurate continuum dielectric calculations to specify the range of p_A_ values consistent with the structural model of the minor state is significantly enhanced.

Since this approach to analyzing transient protein conformations is based upon accepting or excluding specific model conformations, it cannot serve to formally eliminate the possibility that any single structurally consistent Boltzmann-averaged transient conformational state for the β_2_–β_3a_ hairpin of the FKBP51 domain is capable of explaining the differences in hydrogen exchange reactivity observed for the wildtype and K58T variant proteins. Such analyses can only indicate the relative implausibility of such a single transient state. On the other hand, the number of robust experimental measurements within the β_2_–β_3a_ hairpin of these FKBP51 domains are sufficient to usefully constrain the sets of log k_OH−_ values and population values for a pair of transient conformational states that are mutually consistent with predicting the observed wildtype hydrogen exchange reactivity. This extension to a three-state model introduces the need to characterize the fraction of the differential hydrogen exchange reactivity that arises from each of the two minor states. In practical terms, only a subset of the residues being considered are likely to have significant exchange reactivity contributions from both minor states. In the present example, for Lys 60, Leu 61, and Lys 65, the predicted exchange reactivity of the 4TW6-based minor state conformation is either too weak to detect or substantially lower than the reactivity of the wildtype protein and thus incapable of significantly contributing to that value (Table 1). As analyzed in detail later, for the six residues exhibiting significant differential hydrogen exchange, there are nine predicted peptide reactivity values that must serve to constrain the two minor state population values p_A_ and p_B_ as well as three fractional contribution values for the exchange reactivities of the two minor states at Gly 59, Gly 64, and Phe 67.

### Analysis of the 4TW6- and 4TW7-derived conformations in predicting wildtype log k_OH−_ values

As summarized previously, the electrostatic predictions of log k_OH−_ values for the residues of the β_2_–β_3a_ hairpin residues in the 4TW6- and 4TW7-derived conformations are qualitatively compatible with the assumption that the increased hydrogen exchange reactivity of the wildtype protein can be explained in terms of such transient conformations. The testing of that hypothesis can be usefully divided into two distinct questions. Within the estimated precision of the Poisson–Boltzmann electrostatic calculations of peptide acidity, can a Monte Carlo sampling of the 4TW6- and 4TW7-based DelPhi-predicted log k_OH−_ values (Table 1), combined with the experimentally determined hydrogen exchange values for the K58T variant, give rise to predictions of the experimental log k_OH−_ values for the wildtype protein to within their estimated uncertainty? There are nine model log k_OH−_ values (three from the 4TW6-based model and six from the 4TW7-based model) that are anticipated to significantly contribute to the optimal fitting of the wildtype hydrogen exchange reactivity. If sets of model log k_OH−_ values derived from Monte Carlo sampling of the DelPhi-predicted peptide acidities are found to be consistent with the experimental wildtype hydrogen exchange data, can these compatible sets serve to identify a nearby subregion within the nine-dimensional space of model log k_OH−_ values that can be used to assess the statistical robustness of the resultant population predictions for the modeled transient conformations?

For this Monte Carlo analysis, the average uncertainties of 0.040 and 0.046 were applied to the log k_OH−_ values for the wildtype and K58T variant samples, respectively. With regard to the residues in the β_2_–β_3a_ hairpin, with one exception, all the individual experimentally derived uncertainty values from the CLEANEX-PM measurements were less than the assumed average uncertainty values (Table S1). As the CLEANEX-PM measurements for Lys 60 in the wildtype protein yielded an uncertainty value of 0.09, for that residue, the Monte Carlo sampling analysis was applied using this larger uncertainty value. Consistent with earlier estimates in the uncertainty of the Poisson–Boltzmann electrostatic calculations of peptide acidity (33), a Gaussian-distributed uncertainty of 0.5 was applied to the log peptide acidity values for amide hydrogens exposed to solvent in at least 50% of sampled trajectory frames, whereas a value of 0.7 was applied to the amide hydrogens with solvent exposures between 1% and 50%.

For every Monte Carlo sampling that is compatible with the experimental wildtype hydrogen exchange data, the individual predicted transient state log k_OH−_ values must typically lie within a range that is determined by the uncertainty assigned to that experimental exchange data. On the other hand, since the range of the statistical sampling in the Monte Carlo analysis is more than 10-fold larger because of the greater uncertainties of the structure-based electrostatic predictions, the vast majority of the resultant Monte Carlo predictions can be expected to be incompatible with the experimental wildtype hydrogen exchange data. Conversely, these broad sampling distributions also ensure that even initial log k_OH−_ values that are well removed from their optimum will still have an appreciable probability of yielding a compatible Monte Carlo prediction. For the six residues that exhibit significant differential hydrogen exchange kinetics for the wildtype and K58T variant, 30 million Monte Carlo samplings were generated by a Gaussian-distributed modulation of the experimental K58T variant log k_OH−_ values and the nine structurally derived DelPhi log k_OH−_ value predictions. Among these samplings of the three-state model, 7125 (0.024%) yielded predictions of the wildtype experimental log k_OH−_ values to within a χ^2^ value of 6 (equivalent to an rmsd of 0.04) based upon the assumed uncertainties.

**Figure 8:**
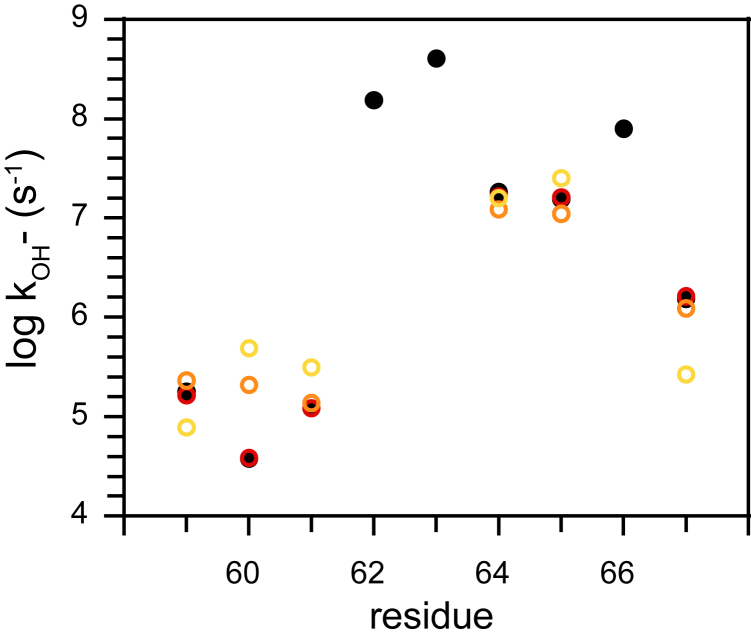
**Predictions of hydrogen exchange reactivities in the wildtype FKBP51 domain for two-state and three-state models utilizing 4TW6- and 4TW7-based structural analysis.** Log k_OH−_ value predictions for the wildtype domain based solely on the 4TW7-based DelPhi electrostatic calculations as applied to a single transient state model (*yellow*) strongly deviate from the experimentally observed log k_OH−_ values (*black*) with a χ^2^ value of 721. Utilization of both the 4TW6- and 4TW7-based electrostatics predictions (*orange*) in the three-state model analysis reduced this discrepancy to a χ^2^ value of 114.7. Following Monte Carlo sampling of the combined 4TW6- and 4TW7-based DelPhi log k_OH−_ values, the median values of the experimentally compatible samplings (*red*) were well within the uncertainty limits of those measurements (χ^2^ of 2.29). FKBP, FK506-binding protein.

Comparison was also made to the alternative two-state single transient model being applied to the observed wildtype hydrogen exchange data. As summarized in Table 1, for every residue of the β_2_–β_3a_ hairpin, the log k_OH−_ value predicted from the iFit4-inhibited protein conformation was larger than the corresponding experimental value of the wildtype protein. On that qualitative basis, a calculation based upon this single conformation might potentially prove adequate to rationalize the wildtype data in terms of the two-state model. However, when the analogous Monte Carlo samplings were carried out using only these 4TW7-derived hydrogen exchange reactivities as reference values, the frequency of predictions that were compatible with the wildtype hydrogen exchange data was 230-fold lower (∼1 in 10^6^) than what was observed utilizing the three-state model based upon both the 4TW6- and 4TW7-based structures. This disparity in the frequency with which experimentally compatible Monte Carlo samplings were obtained from these two-state and three-state models reflect the statistical consistency that is deduced from directly applying the DelPhi-derived log k_OH−_ value predictions (Table 1) to Equation 2 utilizing either the 4TW7-based calculations alone or combined with the 4TW6-based calculations. When the resultant predictions for the wildtype hydrogen exchange kinetics were compared against the experimentally observed values, the disparity, as weighted by the experimental uncertainty of those data, yielded a χ^2^ value of 721 (rmsd of 0.439) for the two-state 4TW7-based DelPhi predicted model and a χ^2^ value of 114.7 (rmsd of 0.175) for the three-state 4TW6- and 4TW7-based DelPhi predicted model (Fig. 8). In contrast, when predictions for the wildtype hydrogen exchange kinetics were drawn from the median residue log k_OH−_ values obtained from the Monte Carlo optimization of the initial DelPhi-predicted values, a χ^2^ value of 2.29 (rmsd of 0.025), well within the experimental uncertainty of these hydrogen exchange reactivity measurements (Fig. 8).

The shifts away from the initial DelPhi-predicted log k_OH−_ values that resulted from the Monte Carlo optimization were dominated by the change in the log k_OH−_ value for Lys 60 in the 4TW7-derived model from 7.86 to 7.11 (Table S4). All other Monte Carlo optimization–derived median log k_OH−_ values were shifted by less than half of their estimated uncertainties. Despite the far more precise prediction of the experimental wildtype hydrogen exchange behavior provided by the median log k_OH−_ values of the Monte Carlo optimization, when scaled against the estimated uncertainties of the original DelPhi electrostatic calculations, a χ^2^ value of only 1.92 was found to separate the initial DelPhi-derived log k_OH−_ value predictions for the transient states from the corresponding log k_OH−_ values obtained from the Monte Carlo optimization procedure (Table S4).

Among the 7125 compatible samplings obtained from the first round of Monte Carlo sampling, only 47 yielded log k_OH−_ values that were as close to the initial DelPhi-predicted values as were the Monte Carlo–optimized values. Furthermore, each of those 47 individual samplings were substantially closer to the Monte Carlo–derived median log k_OH−_ values than they were to the DelPhi-predicted values. Indeed, for all 7125 compatible samplings, only 6% yielded statistical distances that were closer to the original computationally predicted log k_OH−_ values than to the Monte Carlo–optimized values. This distribution of Monte Carlo samplings that are compatible with the wildtype experimental log k_OH−_ values is consistent with the expectation that such compatible log k_OH−_ values will tend to be clustered and that the nearest such clustering lies along the direction between the DelPhi-predicted log k_OH−_ values and the Monte Carlo–derived median log k_OH−_ values. When a second round of this Monte Carlo optimization protocol was performed using the median log k_OH−_ values derived from the first round as input, none of the derived median log k_OH−_ values shifted by more than 0.15. This result suggested that the iterative Monte Carlo protocol had approached a local optimum.

In statistically characterizing the individual compatible samplings from the Monte Carlo optimization of the model log k_OH−_ values, it was immediately apparent that the distribution of the population predictions strongly deviated from normality. Not only did the populations of the 4TW6- and 4TW7-derived transient conformations and their ratio (p_w6_, p_w7_, and p_w6_/p_w7_) each have skew values (measuring the third moment of the distribution) in excess of 2.0, their (excess) kurtosis values (measuring the fourth moment of the distribution) were 5.6, 12.3, and 11.1, respectively. As evident from Equation 2, the source of this severely non-Gaussian distribution of the modeled state populations arises from the fact that the Gaussian-distributed noise is introduced into the model log k_OH−_ values and as a result will modulate the corresponding state population values in an exponentially distributed fashion. When transformed into the log population values for p_w6_, p_w7,_ and p_w6_/p_w7_, each skew value was <±1 and each kurtosis value was <±2. In carrying out the statistical analysis on the log population distribution and then exponentiating the derived parameters, the mean values for experimentally consistent peptide reactivities derived from the first round of Monte Carlo median-derived log k_OH−_ values were 0.078, 0.0023, and 34 for p_w6_, p_w7_, and p_w6_/p_w7_, respectively. These population estimates were altered by less than 10% by the second round of Monte Carlo simulation. As utilized later, this need to carry out statistical analysis on the log population results in the values for the standard deviation being expressed as a ratio.

While the initial round of Monte Carlo analysis of the 4TW6- and 4TW7-based electrostatic calculations appeared to have identified a local optimum for a paired set of model log k_OH−_ values, this interpretation creates the expectation that other sets of log k_OH−_ values, which are within this parameter space neighborhood might similarly tend to migrate toward this set of values upon Monte Carlo optimization. More broadly, can a set of such local optima be identified that generally corresponds to the range of log k_OH−_ values that are consistent with the original DelPhi-predicted peptide acidities to within their estimated uncertainty? Given such a result, do the statistics drawn from that family of compatible cluster optima provide a robust measure of the predicted populations of the two modeled transient conformations? By systematically expanding the range of log k_OH−_ values being analyzed beyond those that are comparatively close to the initially derived DelPhi peptide acidity predictions, the distribution of experimentally compatible log k_OH−_ values can be more fully characterized, and robustness of the corresponding state population predictions can be more completely assessed.

**Figure 9:**
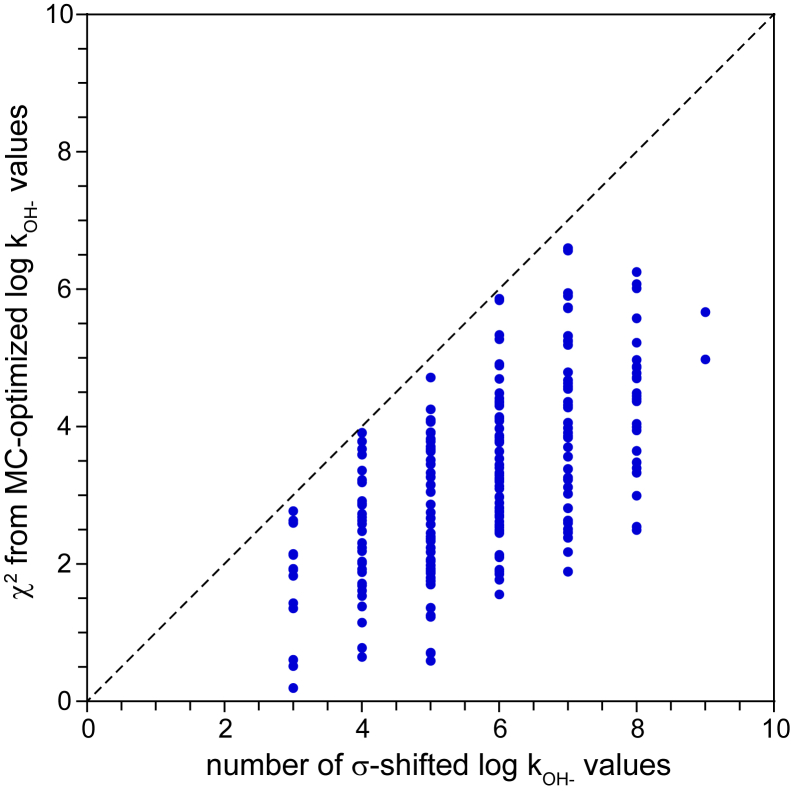
**The statistical distance between the initial Monte Carlo optimized log k**_**OH−**_**values and those resulting from the subsequent σ-shifted Monte Carlo samplings.** Each of the initial Monte Carlo–optimized log k_OH−_ values for the two putative model transient conformations was randomly altered by its computational uncertainty (*i.e.*, ±0.5 or 0.7) so as to generate 250 independent sets that were then subjected to a round of Monte Carlo optimization. Without exception, every set of log k_OH−_ values shifted back toward the initial Monte Carlo–optimized values.

Monte Carlo simulations were carried out on 250 independent sets of model log k_OH−_ values. In every set, each of the nine log k_OH−_ values obtained from the initial round of Monte Carlo optimization were randomly altered at a probability of one-third to be increased or decreased by their assigned uncertainty value (*i.e.*, 0.5 or 0.7) or else left unchanged. Strikingly, in every instance, the median log k_OH−_ values predicted by the Monte Carlo simulations on each of these sets of σ-shifted log k_OH−_ values migrated back toward their unshifted values (Fig. 9), indicating that in some sense, the set of unshifted log k_OH−_ values approximates a local center in the parameter space of experimentally compatible log k_OH−_ values. It should be noted that, given the σ-shifting protocol used, initial χ^2^ values less than 3 (*i.e.*, only 0-, 1-, or 2-residue log k_OH−_ values were σ-shifted) are strongly disfavored so that all sampled sets of model log k_OH−_ values are significantly more distant from the initial Monte Carlo–optimized model log k_OH−_ values than are the original DelPhi-predicted log k_OH−_ values (χ^2^ = 1.92). Indeed, even when as many as seven or eight of the nine log k_OH−_ values have been shifted away from what had been obtained in the initial Monte Carlo optimization of the DelPhi-predicted log k_OH−_ values, there are instances in which the optimization decreased their χ^2^ value by more than 5, far beyond the migration that occurred in the initial Monte Carlo optimization of the DelPhi-predicted log k_OH−_ values.

A second round of Monte Carlo optimization was applied to the sets of σ-shifted model log k_OH−_ values. Among these sets, the average shift in the χ^2^ values for this round of optimization was 0.16 with no apparent bias in further migration toward the initial Monte Carlo optimization of the DelPhi-predicted log k_OH−_ values. Given the modest shifts resulting from the second rounds of Monte Carlo optimization and the desire to minimize the potential effects of statistical overfitting, only the results from the first rounds of Monte Carlo optimization were further considered. The statistical distances between the Monte Carlo optimization of the DelPhi-predicted log k_OH−_ values and various Monte Carlo optimizations of the σ-shifted model log k_OH−_ values yielded an average χ^2^ of 3.3. The dispersity in these positions of local optimization is further indicated by their median pairwise statistical separation of 5.5 with only 2% of the χ^2^ values between pairs of Monte Carlo–optimized σ-shifted model log k_OH−_ values being less than 1.0. Approximately half of these positions of local optimization (52%) yielded a higher frequency of Monte Carlo samplings compatible with the wildtype FKBP51 domain hydrogen exchange data than what was predicted from the initial Monte Carlo optimization of the DelPhi-predicted log k_OH−_ values.

The region of the nine-dimensional log k_OH−_ parameter space that is sampled by the Monte Carlo–optimized σ-shifted log k_OH−_ values covers a range of parameter values comparable to the uncertainty estimates for current methodologies in structure-based prediction of peptide acidities. When the p_w6_, p_w7_, and p_w6_/p_w7_ values derived from the full set of Monte Carlo–optimized σ-shifted log k_OH−_ values were averaged, the values 0.064, 0.0020, and 32 were obtained. The standard deviation ratios for those p_w6_, p_w7_, and p_w6_/p_w7_ values were 1.7, 1.6, and 2.0, respectively, yielding ±1σ intervals of (0.038–0.107), (0.0013–0.0032), and (16–64), respectively. The mean log k_OH−_ values derived from the Monte Carlo optimization of σ-shifted model log k_OH−_ values differ from the original DelPhi-predicted log k_OH−_ values by a χ^2^ value of 2.88 (Table S4). Within this region of the log k_OH−_ parameter space, these results indicate that the population values for any pair of transient conformations, which are capable of jointly predicting the hydrogen exchange behavior in the β_2_–β_3a_ hairpin of the wildtype FKBP51 domain, can be specified to a useful level of precision.

## Discussion

This study has shown that in favorable circumstances, structure-based peptide reactivity analysis can impose powerful constraints upon explaining examples of protein flexibility in terms of either single structure or dual structure models for the indirectly inferred transient states. In the particular application considered, it has been found that the hydrogen exchange behavior in the β_2_–β_3a_ hairpin of the unliganded FKBP51 domain is consistent with that region transiently adopting two distinct conformations that closely resemble those seen in the iFit1- and iFit4-inhibited forms of this protein. Given the 32-fold difference in the predicted populations of these two transient conformations (0.064 and 0.0020, respectively), this ratio would indicate a 2.1 ±0.4 kcal/mol difference in the stability of these two conformations in the unliganded protein. In a strict conformational selection perspective, the differences in inhibitor-binding interactions can be analyzed as a tradeoff between the reduced accessibility of the iFit4-like conformation and an accompanying enhanced strength of binding interaction for that transient conformation. Alternatively, from the perspective of an induced fit mechanism, the weaker binding but more readily accessible iFit1-like conformation might serve as an initial site for inhibitor interaction that then facilitates the transition to a more stable iFit4-like complex.

**Figure 10:**
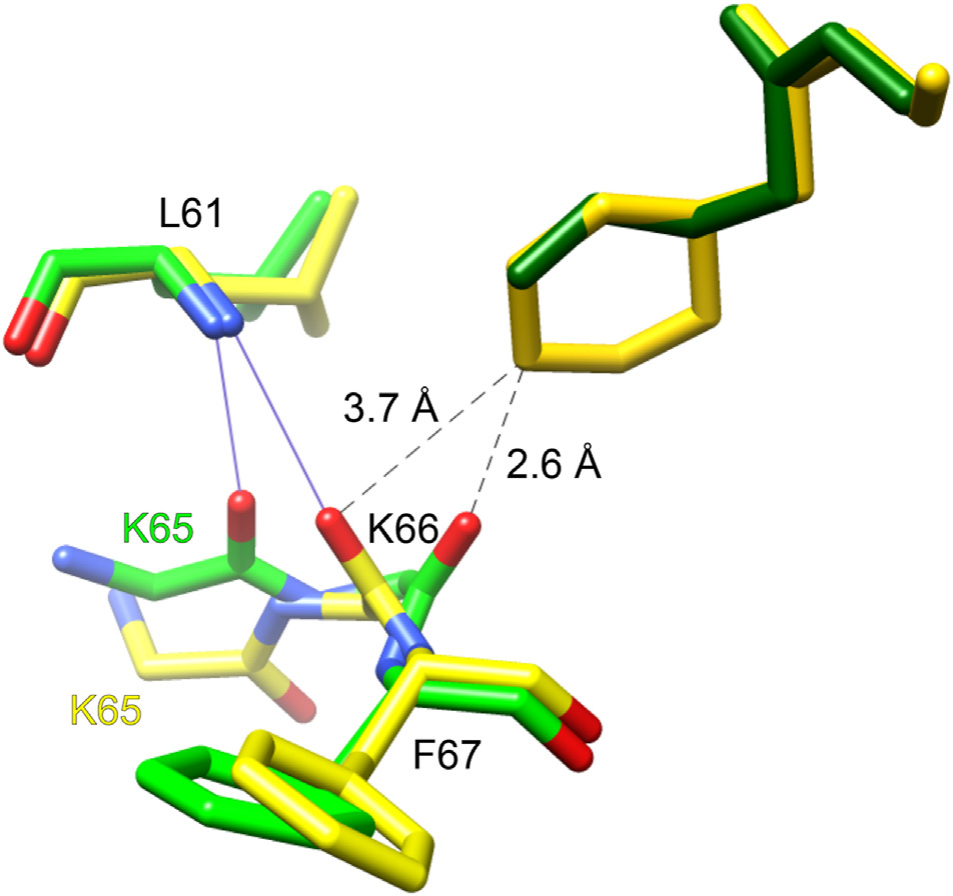
**Structural superposition of the inhibitor–protein interactions in the β**_**2**_**–β**_**3a**_**hairpin region of the iFit1- and iFit4-bound complexes.** In contrast to the smaller allyl group of the iFit1 inhibitor (*dark green* at *upper right*), the positioning of the larger cyclohexene ring of the iFit4 inhibitor (*gold*) is incompatible with the orientation of the carbonyl group of Lys 66 as seen in the iFit1-bound structure (*green*). The shifting of that carbonyl group in the iFit4-bound structure (*yellow*) is accompanied by a hydrogen bonding to the amide of Leu 61 and a flip of the peptide group linking Lys 65 and Lys 66.

Potential insight into this question can be gained from considering the differing interactions of the FKBP51 domain with the iFit1 and iFit4 ligands as visualized in the superposition of the 4TW6 and 4TW7 crystal structures. The cyclohexene ring of the iFit4 inhibitor is well established to provide the central role in defining the differential binding interactions that distinguish the iFit4-derived series of lead compounds as compared with the iFit1-related inhibitors. Through an extensive series of analog studies, the cyclohexene ring has been found to be strikingly sensitive to even minor modifications, blocking all efforts at replacement until the recently reported substitution of the sterically similar thiophene ring (52). In the upper right corner of Figure 10 is seen the cyclohexene ring of the iFit4 inhibitor as well as the much smaller allyl group of the iFit1 inhibitor. While the iFit1 allyl group exhibits favorable steric interactions with the protein backbone of the β_2_–β_3a_ hairpin in the 4TW6 conformation, the superimposed cyclohexene ring of the iFit4 inhibitor would form a severe steric overlap with the carbonyl oxygen of Lys 66 as positioned in the iFit1-bound state (heavy atom distance of 2.6 Ǻ). This apparent overlap is relieved in the 4TW7 structure by a pivoting of the Lys 66 carbonyl group, which enables it to form a hydrogen bond to the amide of Leu 61. Concurrently, the hydrogen bond between the carbonyl oxygen of Lys 65 with the amide of Leu 61 that is present in the 4TW6 structure is disrupted in the 4TW7 structure as the peptide group connecting Lys 65 and Lys 66 is flipped by ∼180°. It should be noted that this flipping of the Lys 65–Lys 66 peptide group has the effect of removing the locally unfavorable energetics of a positive ϕ value at Lys 66 ([ψ_65_, ϕ_66_] from [165°, 56°] in 4TW6 to [86°, −142°] in 4TW7). As the unliganded FKBP51 domain has a comparatively low energy conformation at this position, the initial transition to the iFit1-like conformation could be seen as generating a localized site of stress around the Lys65–Lys 66 peptide linkage. In such a scenario, the initial interaction of the iFit4 inhibitor with the transiently exposed iFit1-like conformation might then trigger a second smaller scale transition in the protein to generate a more tightly binding conformation.

As reflected in the naming of their selective inhibitor series, Hausch *et al.* (9) initially proposed an induced fit mechanism for these binding interactions. Evidence for a protein conformational transition occurring at the inhibitor binding site in the absence of the inhibitor prompted the counterproposal of a conformational selection mechanism by both our group (11) and theirs (14). The present evidence for two distinct transient conformations in the unliganded FKBP51 domain arguably establishes a useful model system for a more detailed comparison between the induced fit and conformational selection mechanisms for drug interactions. One potentially promising approach to systematically altering the binding behavior for either the iFit1-like or iFit4-like conformations of FKBP51 is a recently described high-throughput yeast display screening analysis for this protein utilizing both random mutagenesis and site saturation mutagenesis to generate variants with altered affinities for an iFit4-like fluorescently labeled ligand (53). The five reported variants that exhibited a binding constant for the fluorescent ligand that was more than twofold above that for the wildtype protein were all drawn from residue positions 63, 64, and 67. While further studies would be needed to quantify the degree to which those enhanced interactions reflect altered binding interactions with the ligand as opposed to an increased population of a ligand-favorable protein conformation, such mutational variants might usefully modulate either the binding affinities or binding kinetics so as to provide further insight into optimizing structure-based design approaches when protein conformational transitions both before and after the initial ligand interaction must be taken into account.

## Experimental procedures

### Protein expression and purification

Genes for the wildtype and K58T variant of the human FKBP51 FK1 domain (Glu 20–Glu 140) were chemically synthesized (Genscript) with codon optimization for expression in *Escherichia coli* (22). The genes were cloned into the expression vector pET11a and then transformed into the BL21(DE3) strain (Novagen) for expression. The [^2^H,^15^N]-labeled proteins were expressed and purified as previously described (8). For the CLEANEX-PM magnetization transfer experiments, aliquots of the [U-^2^H,^15^N]-enriched protein were concentrated by centrifugal ultrafiltration and then exchanged into a series of buffers containing 6% ^2^H_2_O in which the buffer concentration for each pH value was set to 25 mM, with reducing agents dithiothreitol and Tris(2-carboxyethyl)phosphine hydrochloride at 1 mM, and sodium chloride was added to a final ionic strength of 150 mM. For these experiments, a set of six pH values were collected for each protein (6.03, 6.50, 7.41, 8.47, 9.31, and 9.68 for the wildtype FKBP51 and 5.99, 6.48, 7.46, 8.43, 9.32, and 9.72 for the K58T variant). Sodium phosphate buffers were utilized for the three lowest pH samples, sodium borate buffers were utilized for the next two higher pH values, and a sodium carbonate buffer was utilized for the highest pH value. For the hydrogen–deuterium exchange experiments on the K58T variant, an exchange-in protocol was used (25). In contrast to the more conventional exchange-out protocol, the protein sample was initially back-exchanged for 3 h at 25 °C in ^2^H_2_O-containing buffer near a p^2^H of 9 and then equilibrated to the desired final p^2^H of approximately 6.5 with a phosphate buffer composed as described for the CLEANEX-PM experiments. The sample was then concentrated and lyophilized. The CT-CPMG experiments were carried out on protein samples in which the slowly exchanging peptide hydrogens were back-exchanged to protons by a similar high pH incubation. The sample was then neutralized and equilibrated into a 25 mM sodium phosphate buffer (pH 6.50) containing dithiothreitol and Tris(2-carboxyethyl)phosphine hydrochloride at 1 mM.

### NMR data collection

All experimental NMR data were collected on a Bruker Avance III 600 MHz spectrometer equipped with a TXI cryoprobe at a probe temperature of 25 °C with samples at protein concentrations of ∼0.5 mM. Utilizing our previously reported ^1^H, ^15^N, and ^13^C resonance assignments for the FKBP51 domain protein (Biological Magnetic Resonance Bank entry: 19787 (8)), CLEANEX-PM (38) spectra were obtained. For each sample, a series of four CLEANEX-PM experiments were collected with mix times of 6.49, 11.68, 21.41, and 38.93 ms using the *fhsqccxf3gpph* Bruker pulse program. Interleaved with these spectra were a complementary set of reference hard pulse spectra in which the e-PHOGSY selective water excitation pulse (39) was replaced with a high power 90° pulse to enable postacquisition compensation for the relaxation effects that give rise to nonlinearity in the CLEANEX-PM buildup curves (40). Taking advantage of the increased intensity of the hard pulse reference spectra, four scans per t_1_ increment were collected as compared with 16 scans per t_1_ increment that were used in the CLEANEX-PM spectra. The indirect dimension was collected to a t_1_ maximum of 0.0548 s. Recycle delays of 4.0 s were used in all experiments except for an additional repeat of the shortest mix time hard pulse experiment that utilized an 8.0 s recycle delay, which was used to estimate the fully relaxed peak intensities. Processing of the spectral data was carried out with Felix software (Felix NMR).

For the ^1^H exchange-in experiments, the lyophilized K58T variant sample was dissolved in 94% ^1^H_2_O–6% ^2^H_2_O, and a time series of ^1^H–^15^N 2D transverse relaxation optimized spectroscopy (54) spectra was collected, yielding spectral quality as illustrated by Leu 61 (Fig. S2). In contrast to hydrogen exchange measurements carried out in deuterated water, there is no deuterium isotope–induced shift either in the ionization behavior or in the protein stability (55). The only significant differential isotope effect that arises during the ^1^H exchange-in experiments is the 0.08 unit shift in the log rate constants for exchange at N–^2^H amide bonds, relative to the corresponding N–^1^H bonds (56). A CLEANEX-PM experiment was collected on the exchange-in sample, which in comparison to the previously analyzed pH series of CLEANEX-PM datasets provided a pH value of 6.50. Since in no instance did the measured log k_OH−_ values for the K58T variant residues 59, 60, 61, and 67 contribute more than 25% of the predicted wildtype exchange rate, the modestly reduced experimental uncertainty of those data was of minimal influence on the resultant analysis.

The ^1^H^N^ CT-CPMG relaxation dispersion experiments were carried out using an 80 ms CT interval and a recycle delay of 5.0 s between scans (41). Sixteen scans per t_1_ increment were collected to a t_1_ maximum of 0.0740 s. In addition to a hard pulse reference experiment, a series of 24 CT-CPMG experiments were collected with the spacing between the 180° ^1^H pulses being altered so as to yield refocusing frequencies of 50, 125, 250, 425, 650, 925, 1250, 1600, 2000, 2400, 2800, 3200, 4000, 440, 4800, 5200, 5600, 6000, 6400, 6800, 7200, 7600, and 8000 Hz collected sequentially. Two complete relaxation dispersion datasets were collected on both the wildtype and K58T variant samples.

### Hydrogen exchange analysis

For each mix time, the amplitude for every CLEANEX-PM crosspeak was normalized by the fractional signal loss because of the selective PHOGSY pulse sequence component and the relative number of scans used in the CLEANEX-PM and the hard pulse reference spectra. The normalized CLEANEX-PM intensity was then subtracted from the amplitude of the corresponding hard pulse reference crosspeak normalized to its fully relaxed intensity to yield an exponential decay with time constant ρ + *k*_ex_. Utilizing Equation 1, values of *k*_ex_•t were derived for each mix time. Under the assumption of a consistent peak intensity noise level, the uncertainty of the resultant slope was calculated (57):

(3) $$\sigma_{slope}=\left(\frac{1}{n-2}\right)\left[\sum_{i-1}^{n}{\left(y_{i}-{ŷ}_{i}\right)}^{2}\right]/\;\left[\sum_{i=1}^{n}{\left(x_{i}-\bar{x}\right)}^{2}\right]$$

where *x̅* is the average of the CLEANEX-PM mix times and ŷ_i_ is the value of the best fit line at each mix time.

For the large majority of residues, the derived log exchange rates were found to vary linearly with the sample pH. Two factors can complicate this straightforward analysis. The line-broadening relaxation effects that arises from conformational dynamics in the ∼millisecond time frame can introduce a pH-independent contribution to the observed CLEANEX-PM signal. Measurements at multiple pH values can be used to deconvolute the hydrogen exchange contribution from the conformational exchange contribution. For the circumstance in which the conformational exchange contribution is comparatively modest, as with the residues Gly 59 through Phe 67 analyzed in this study, the log k_OH−_ value derived from the highest pH monitored agreed to within 0.1 with the value deduced from a fitting of the exchange data assuming a pH-dependent and pH-independent component across the pH values monitored. In contrast, residues in the β_3a_–β_3b_ loop exhibit the combined challenge of larger conformational line-broadening effects and the titration of His 71 side chain, which introduces the complication of presenting two distinct electrostatic environments for the nearby backbone amides. As a result, the accurate quantitation of the log k_OH−_ values was compromised for a number of these residues.

### Peptide acidity calculations

Using the visual molecular dynamics program (58), the X-ray coordinates of the unliganded protein and crystallographic waters (PDB codes: 4TW6 and 4TW7 (9)) were imbedded in a water box with a minimum distance of 10 Å from the protein atoms to the nearest boundary. Visual molecular dynamics was used to add 19 Na^+^ and 21 Cl^−^ ions for an ionic strength of 150 mM, similar to the experimental conditions. Energy minimization and stepwise heating to 298 K was applied in the NAMD2 simulation package (59) with the CHARMM36 force field (50) and particle mesh Ewald electrostatics using a 64 × 64 × 64 grid. Equilibration of the solvated protein was carried out as previously described (33). Production runs of 50 ns were carried out under NVE conditions. A 1 fs timestep was applied with switch distance, cutoff, and pairlist distances of 10 Å, 12 Å, and 14 Å, respectively.

Poisson–Boltzmann continuum dielectric calculations were carried out on the trajectory frames sampled at 50 ps intervals from each of the molecular dynamics simulations as previously described (25, 60). The SURFV program (61) with the default set of atomic radii (62) was used to determine the static solvent accessibility. The DelPhi program (51) was used for linear Poisson–Boltzmann predictions of the electrostatic potentials for each solvent-exposed backbone amide in each structure from the sampled trajectory frames using the CHARMM22 atomic charge and radius values (63). Based upon previous studies (60), a minimum value of 0.5 Ǻ^2^ exposure to the solvent phase was applied. The electrostatic potential calculations were carried out utilizing a 50% fill factor, a 0.4 Ǻ grid spacing, an ionic strength of 0.15 M, and an ion radius of 2.0 Ǻ.

Internal and solvent dielectric values of 3.0 and 78.5 were applied. The empirical robustness of this internal dielectric constant value for peptide acidity calculations has previously been demonstrated on both model peptides and protein crystallographic structures (22, 28). The differential charge distribution applied to each peptide anion was previously derived from *ab initio* modeling of *N*-methylacetamide and the *N*-methylacetamide anion with MP2 and B3LYP (64) density functional theory calculations using Gaussian03 (65) initially at the aug-cc-pVDZ basis set level *in vacuo* and with polarizable continuum model solvation at dielectric values of 3 and 78.39 with subsequent optimization at the aug-cc-pVTZ basis set level (22). Based upon those calculations, the excess negative charge of the peptide anions has been distributed as −0.09, −0.17, and −0.54 on the C, O, and N atoms, respectively, with an additional differential charge of −0.10 being placed upon each adjacent C^α^ atoms. All protein peptide anion electrostatic potentials were calculated relative to an internal *N*-methylacetamide anion. For each sampled trajectory frame, an *N*-methylacetamide molecule is placed within the DelPhi solvent box with all atoms being at least 8 Ǻ from the nearest protein atom and at least 16 Ǻ between the *N*-methylacetamide nitrogen and the nearest formal charge on the protein so as to minimize the predicted differential electrostatic solvation free energies to less than 0.1 kT (22, 66).

For each backbone amide that exhibited solvent exposure in at least 1% of the sampled frames, the derived peptide kinetic acidity values were averaged across the trajectory in which the diffusion-limited rate was attenuated by the factor *K*_i_/(*K*_i_ + 1), where *K*_i_ is the equilibrium constant for the transfer of a hydrogen from the amide to the hydroxide ion. The internal scaling of the effective p*K*_a_ of water for each trajectory run was carried out by minimizing the difference between the predicted and experimental log k_OH−_ values for the residues that exhibited solvent exposure in more than 50% of the sampled frames, excluding the first six residues and positions Gly 59 to Ile 87. The rmsd values for the difference between these predicted and experimental log k_OH−_ values averaged over the four independent 50 ns molecular dynamics trajectories were 0.57 and 0.61 for the 4TW6- and 4TW7-based models, respectively. Figures of crystallographic structures were generated using CHIMERA software (UCSF Resource for Biocomputing, Visualization, and Informatics at the University of California) (67).

### Relaxation dispersion analysis

Relaxation dispersion analysis was carried out utilizing the RELAX analysis program (version 5.0.0 (45)) (NMR spectroscopists; http://www.nmr-relax.com). Exclusion was set for datasets in which the apparent R_2(eff)_ was less than 2 s^−1^. For each ^1^H^N^ CPMG data series, uncertainty estimates for peak intensities were derived from pooled rmsd values obtained from intensity measurements performed on repeated spectra. Five hundred rounds of Monte Carlo samplings were applied for each dataset. Each dataset was analyzed both with and without inclusion of the Carver–Richards dynamical model. In every instance for which relaxation dispersion was identified, all residues outside the β_4_–β_5_ loop were statistically assigned to the simpler Luz–Meiboom dynamical model. Within the β_4_–β_5_ loop, four residues in the wildtype domain and three residues in the K58T variant domain were assigned by the Akaike information criterion to the Carver–Richards model in both datasets collected. One other residue in the wildtype domain and three other residues in the K58T variant domain yielded differing model assignments between the two repeated dataset collections.

## Data availability

All discussed experimental data are included in the article and/or the supporting information. Raw NMR data are available upon request.

## Supporting information

This article contains supporting information.

## Conflict of interest

The authors declare that they have no conflicts of interest with the contents of this article.
