# Supplementary material for: Transient conformations in the unliganded FK506 binding domain of FKBP51 correspond to two distinct inhibitor-bound states
Source: J Biol Chem. 2023 Aug 12;299(9):105159. doi: 10.1016/j.jbc.2023.105159 (PMC10514456; doi:10.1016/j.jbc.2023.105159)
Supplement: Supplemental Figs. S1, S2 and Table S1 [file mmc4.pdf]

Fig. S1 – CLEANEX-PM spectra for Val 40 of the wild-type FKBP51 domain

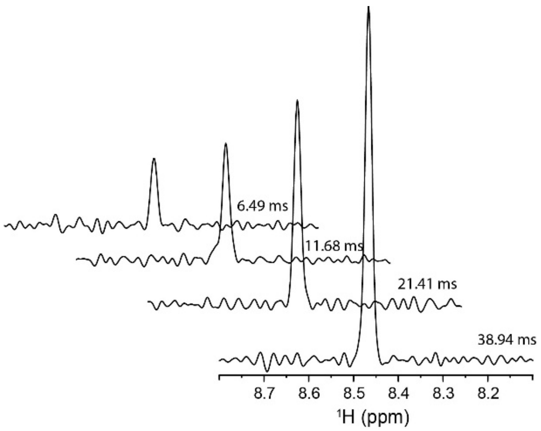

The 1D traces from differing mix times for the CLEANEX-PM $^1H^N$ crosspeak of Val 40 at pH 8.47.

Fig. S2 – $^{1}H^{N}$ exchange-in spectra for Leu 61 of the K58T variant domain of FKBP51

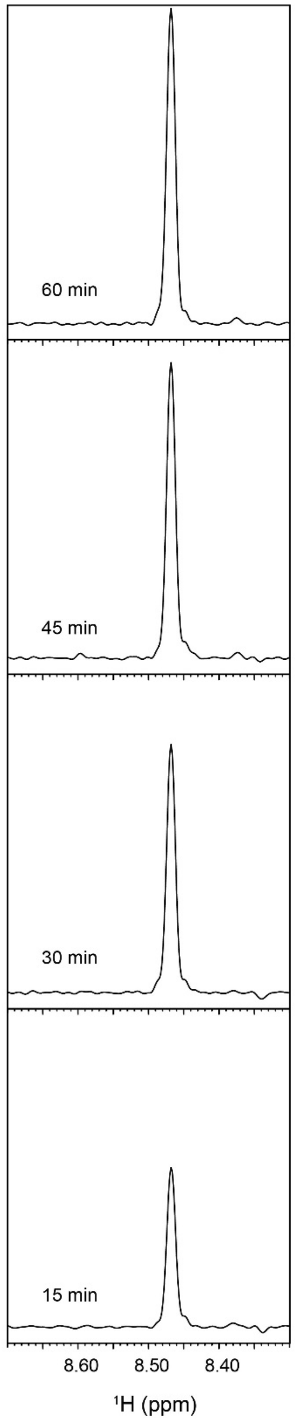

The 1D traces from the first four time series spectra for the $^{1}H^{N}$ crosspeak of Leu 61 at pH 6.50.

Table S4. Log k_{OH-} values consistent with wild-type FKBP51 domain hydrogen exchange

	DelPhi-predicted[a]		MC-opt of DelPhi[b]		MC-opt of σ-shifted[c]	
	4TW6	4TW7	4TW6	4TW7	4TW6	4TW7
GLY 59	5.98	6.73	5.93	6.85	5.99	6.97
LYS 60	3.67[d]	7.86	3.67	7.11	3.67	7.10
LEU 61	-----	7.64	-----	7.65	-----	7.65
SER 62	7.39	8.71	7.39	8.71	7.39	8.71
ASN 63	8.99	9.51	8.99	9.51	8.99	9.51
GLY 64	7.01	9.02	7.32	9.33	7.40	9.39
LYS 65	-----	9.66	-----	9.81	-----	9.82
LYS 66	8.33	8.71	8.33	8.71	8.33	8.71
PHE 67	6.76	7.51	7.00	7.59	7.15	7.59

[a] Poisson-Boltzmann predicted peptide acidities for β_2-β_{3a} hairpin

[b] Median values of Monte Carlo samplings from DelPhi-predicted log k_{OH-} values

[c] Median values of Monte Carlo samplings from σ-shifted log k_{OH-} values

[d] Positions not anticipated to influence log k_{OH-} values predictions remained fixed
